# Foreign Summary

**Published:** 1838-10-24

**Authors:** 


### FOREIGN SUMMARY.

In the London Medical Gazette for July, we find a new mode of administering colchicum in rheumatic gout, which we think may prove important. We often gain more by discovering a new and useful method of administering an old remedy, than by introducing one yet untried. We have seen so many modes of treating the acute rheumatic affections prove more or less incapable of subduing the disease, that we are desirous of testing the value of new modes of treatment, provided they come well recommended. The present mode of giving colchicum, comes from Mr. Wigan, a respectable practitioner of Brighton:

"The powdered root of colchicum is, then, the specific on which I depend. It is an old remedy, but the mode of administration is new, and entirely my own. If the bowels be loaded, I begin by an enema of decoct. aloës, but this does not delay the use of the colchicum. The dose, eight grains every hour, taken in the medium most acceptable to the patient—plain water, sugar and water, apple tea, ginger tea, or other analogous fluids, changing them from time to time, even with the successive doses, if necessary, according as the stomach is more or less irritable, or the palate more or less capricious. The point of saturation, as I call it, (for want of a better term,) is very uncertain, varying so much with different individuals, that although the usual quantity is eight or ten doses, I have known some take fourteen, and others unable to bear more than five. In every case it is to be repeated till active vomiting, profuse purging, or abundant perspiration take place; or at least till the stomach can bear no more. If a slight nausea comes on after three or four doses, (I have never seen it so soon as the fourth,) a quarter of an hour's delay may be allowed. A lump of sugar, dipped in brandy or eau de Cologne, a wine-glass of soda-water, or any thing else the patient wishes for, in small quantity, may be given. Sometimes a small slice of lemon kept in the mouth will turn away the nausea, and enable him to bear a few more doses; the main object in all cases being to get into the stomach the largest quantity that it can be induced to receive. Even two doses taken at once would be rejected by a patient, who will thus gradually bear a dozen. The most usual course of things is this. At the end of the sixth or seventh dose a slight nausea comes on. By keeping quite still, turning away the thoughts by conversation, or listening to an amusing book, coaxing the palate with a slice of lemon, a clove, or some such thing, three or four more doses can be received, when the disgust becomes, perhaps, unconquerable. After this there is generally sound sleep, with occasional nausea on waking. The pain ceases, but the more active effects of the colchicum do not take place for some hours after the last dose. Distressing as is the state of the patient when under the full influence of the medicine, it still does not exceed an ordinary sea-sickness; and when this has been endured for a few hours, it is succeeded by the elysium. The inflammation of the joints subsides, and they resume their natural size with miraculous rapidity. The acidity of the perspiration ceases, as well as the peculiar odour, which enables the experienced practitioner to recognise the disease on entering the room, before he has asked a single question. As soon as a cup of souchong tea can be retained, a sound sleep comes on, from which the patient awakes perfectly well. When circumstances will admit of it, I prefer to give a breakfast of bread and butter and tea only, very early in the morning, and two hours afterwards commence the colchicum. No more food will be required that day, but tea may be given abundantly, with bread sopped in it, if required. It will be well to indulge the returning appetite very sparingly on the day following, on which, however, we may allow a small snap of devilled meat, and rice boiled plain as for curry, which will generally be the things most acceptable to the stomach. A small quantity of good curry itself is not objectionable to those who have been accustomed to that luxury. In the subsequent treatment I have no reason to think that any precaution is necessary. The patient may resume his ordinary diet as soon as the appetite dictates. I have never seen a relapse.

"The colchicum, it is obvious, should be preserved with care. The best mode, I believe, is to grind it, at the proper season, with twice or thrice its weight of fine sugar, into an impalpable powder, when, should it accidentally become damp, it is safe from injury.

"It is better, if possible, that the patient should not be aware of the direct effects expected until they take place, in order that the imagination may not anticipate and interfere with the process."

---

*Researches on the Causes of Abortion.* By MADAME BOIVIN.—Madame Boivin, the distinguished *sage-femme* of Paris, Doctor of Medicine, and chief inspectress of the Royal Maison de Santé, &c., has recently published some excellent remarks on "One of the most frequent and least known causes of Abortion." Her researches have been evidently conducted with great skill and fairness, alike in the examination of the symptoms during life, and of the morbid appearances found on dissection. We cannot do better than report two or three of her illustrative cases, for the purpose of pointing out the nature of the *cause*, which in her opinion gives rise so frequently to miscarriages.

"Madame Kall, twenty-seven years of age, mother of three children, was seized with pleuritic symptoms after exposure to cold. Miscarriage came on: the fœtus was about five months old. She died on the tenth day after the commencement of her illness.

*Dissection.*—(We shall confine our remarks to the state of the uterus and its appendages.) The broad ligaments, the Eustachian tubes, and the ovaries, were grouped or matted together, and adhered to the posterior surface of the uterus. The adhesion was so close, as to require the use of the scalpel to sever it. In the conglomerated mass numerous small tubercles, varying in size from a millet-seed to a pea, were discovered.

It is evident that, *such being the condition of the uterine appendages, the womb could not expand and develope itself, but with extreme difficulty.* Abortion must therefore have come on, although no affection of the chest had supervened. The uterine ligaments being morbidly affected could not readily expand and yield to the gradually increasing distention of the womb: their resistance became the cause of excitation, and a miscarriage was the consequence."

From these short details our readers will be able to understand the reasoning of Madame Boivin, as respects *one of the most frequent and least known causes of abortion.* She adduces many other examples to prove the existence of some morbid change of the uterine appendages in cases of miscarriage. This change, whatever may be its character or appearance on dissection, seems to be almost always the result of some slow, unhealthy inflammation. In some cases, the agglutination of the uterine appendages is found associated with the formation of purulent deposits between the uterus and rectum. Such cases usually occur in scrofulous subjects.

It may now be asked, is there any method of ascertaining, or even of suspecting, the existence of a morbid change in the uterine appendages during life? and, if so, are there any remedial means to remove it?

We must confess that the diagnosis of such a disease is always most difficult and uncertain. The following symptoms may, however, be mentioned as, to a certain degree, indicative of its presence. Menstruation is usually attended with severe pain and suffering; there is often a continual bearing-down and sense of dragging as well during menstruation as during micturition; the evacuation of the bowels too occasions the same feeling; there is often an acute or a dull oppressive pain in one or both groins, extending upwards to the loins and downwards to the limbs; and there is always more or less of leucorrhœal discharge.*

With respect to the treatment of such cases, Madame Boivin informs us that the local use of mercury, the inunction of the mercurial ointment on the groins, thighs, &c., continued so long as gently to affect the system, will often succeed, not only in restoring the health of the patient, but also in removing the tendency to subsequent miscarriages.

Madame Boivin does justice to Dr. Granville of

---

* There is one very important symptom to be ascertained by manual examination, which especially merits the attention of the physician,—we allude to the frequent partial displacement of the neck and orifice of the uterus, and to the *diminution of its natural mobility*, when pressure is made on it by the finger.

The following remarks by Madame Boivin appear to us highly interesting:—"As in the morbid affection of the uterine appendages it is rare that the womb retains its normal degree of mobility, it is therefore of great importance in all cases of irregular menstruation, or of obstinate leucorrhœa, to determine if the womb, independently of the healthy condition of its orifice, preserves all the freedom of movement which results from the normal state of its ligaments, and of the ovaries and Eustachian tubes."

London, in giving him the credit of having suggested this practice in his "Obstetrical Report of the Westminster Dispensary," published many years ago. Dr. G. adduced several cases, which very satisfactorily proved that the tendency to miscarriages may very often be checked by the *mercurial treatment*, in the way of inunction.

The other remedy, recommended by Madame Boivin, is the hydriodate of potash.—*Med. Chir. Rev., from Recherches sur l'Avortement, par Madame Boivin.*

---

*Microscopical Researches on the Composition of the Vaccine Fluid.* By M. Dubois, of Amiens.—[This is a memoir read before the Royal Academy of Medicine: we can only find room for the concluding part of it, which gives the summary of the results obtained.]

1st. The vaccine virus, whether liquid or dry, shows no signs of globules.

2d. The same virus, examined by means of the strongest magnifiers, shows no traces of animalcules.

3d. In its recent state, (that is to say, during the first hours that follow its removal from the pustule,) this virus is remarkably fluid and limpid: by degrees it takes a more solid form and shows a kind of crystallization.

4th. In its state of desiccation, we observe two orders of physical arrangement: viz., 1, lengthened lines, both opaque and transparent, and very slightly interlacing; and 2, a very minute net-work.

5th. These arrangements are essential to good vaccine lymph, and show themselves in all cases exactly in the same way.

6th. When these appearances of the virus are not to be found, the vaccine has lost its contagious properties.

7th. These material conditions may fail either from an anomalous developement of the pustules, and consequently from a constitution previously vitiated, or from accidental causes.

8th. A high and low temperature (ebullition and congelation) hinder the establishment of these physical arrangements.

9th. When the vaccine virus, through the operation of these causes, has not been able to form itself in this way, it loses its contagious properties.

10th. It is not by killing the animalcules that a high and low temperature destroy the properties of vaccine, but by altering its material conditions.

11th. Microscopic examination of the fluid can assist in determining the existence or non-existence of its preservative properties.—*Bull. de l'Acad. Roy. de Med.*, 30 *Avril*, 1838.

---

*A Mode of relieving Patients labouring under Enlargement of the Veins of the Testicle.*—When cases of varicocele, are allowed to proceed without any active means being adopted for their relief, the patients may experience much inconvenience from pain in the loins and spermatic cord, and frequently are incapacitated from walking any considerable distance.

P. W., aged nineteen, applied to me in the year 1832, in consequence of a circocele of very large dimensions, which had existed two years, and had been progressively getting worse. The veins were distended to the size of a large apple; so much was he inconvenienced, that a walk of half a mile produced great pain in the back and spermatic cord. After a consultation with Sir Astley Cooper, cold lotions, suspensory bandages, &c., having been employed without affording the slightest relief, Sir Astley recommended the removal of a portion of the scrotum. To this proceeding the patient would not consent. I therefore adopted the following mode of treatment:—A ring, about an inch in diameter, made of soft silver wire, of a suitable thickness, was padded, and covered with wash-leather. Through this I drew the lower part of the scrotum, whilst the patient was in the recumbent position, and the veins comparatively empty. I then pressed the sides of the instrument towards each other with sufficient force to prevent the scrotum escaping. The use of this instrument every morning before the patient rose from his bed, enabled this gentleman to walk nineteen miles on the third day after the first application; and although he has for six years worn an instrument of this description, he has never experienced the least inconvenience. And I may add, that other patients, (and amongst them medical friends,) labouring under varicocele, have found the greatest relief from this simple contrivance.—*Lond. Med. Gazette.*

---

*Dilatation and Contraction of the Pupil after Death.*—In the report of an autopsy of a case of hydrophobia, furnished in the Edinburgh Medical and Surgical Journal, it is stated: "That a phenomenon, worthy of attention, which has not yet been observed, so far as known to the reporter, in this disease, occurred when examining the *eyes. The iris exhibited the same motions as in life.* The pupil dilated itself, on covering the eye with the eyelids, and again contracted as soon as the light was admitted. These alternated motions were as lively as during life. The colour of the iris was not changed; it was of blue gray, and had only acquired a lustre or brilliancy, which might be called phosphoric. This sensibility of the iris was excited many times, and more than twelve hours after death."

---

*Adulteration of Opium.*—A large quantity of adulterated opium has, it is said, been recently imported from England to Havre, and thence conveyed to Paris. The fraud was fortunately detected by the circumstance of about 120 pounds having been sold to the Pharmacy attached to the Parisian Hospitals. The director immediately detected the fraud, and gave information to the proper authorities, and the whole of the adulterated material was seized at the stores of three wholesale druggists. This opium bears the strongest resemblance to the pure opium of Smyrna. Its taste is analogous to that of good opium, but its smell is a little weaker; neither the watery nor the alcoholic solutions are precipitated by ammonia. This opium presents a characteristic which has hitherto been regarded as a mark peculiar to the genuine drug, viz., the presence of transparent thin layers in the vertical section.—*Jour. de Pharm. July*, 1838.

